# Supplementary material for: Content-rich biological network constructed by mining PubMed abstracts
Source: BMC Bioinformatics. 2004 Oct 8;5:147. doi: 10.1186/1471-2105-5-147 (PMC528731; doi:10.1186/1471-2105-5-147)
Supplement: Additional File 2 — The original results of the above study (non-essential files are deleted to keep the file size under the limit set by BMC bioinformatics). [file 1471-2105-5-147-S2.bz2 › chilibotAdditionalFile2/dip20/350ID8649415E4594/html/IL3_IL3RA.html]

 


 **IL3** and **IL3RA** 
  
Found 22 abstracts in PubMed, retrieved 20.  
 

 What does Google say? 
 PDF only 
| .edu only 

---

**Interactive relationship** (e.g. stimulation, inhibition, etc)

**Neutral relationship**- Freshly isolated CD123  [ **IL3RA** ]  pre DC showed typical plasmacytoid morphology and when cultured with IL 3  [ **IL3** ]  and CD40L for 72 hr, developed mature DC morphology.  Ref: 12829599 Blood< MedlineTA>Blood,
- Single CD34 cells from the CD123  [ **IL3RA** ]  subsets were cultured in a cytokine cocktail of stem cell factor, interleukin 3  [ **IL3** ]  IL 3  [ **IL3** ] , IL 6, GM CSF, erythropoietin, insulin like growth factor 1, and basic fibroblast growth factor.  Ref: 10527461 Stem Cells, 1999

**Non-interactive relationship** (e.g. studied together, co-existance, homology, etc.)

- Interleukin 3  [ **IL3** ]  receptor alpha chain CD123  [ **IL3RA** ]  is widely expressed in hematologic malignancies.  Ref: 11726317 Haematologica, 2001
- Phosphorylation was not detected in response to IL 3  [ **IL3** ]  stimulation, thereby suggesting CD123  [ **IL3RA** ]  is not active in conventional IL 3  [ **IL3** ]  mediated signaling.  Ref: 11021753 Leukemia, 2000
- CD123  [ **IL3RA** ]  alpha subunit of IL 3  [ **IL3** ]  receptor expression on primitive and committed human hematopoietic cells was studied by multicolor sorting and single cell culture.  Ref: 10527461 Stem Cells, 1999
- Two major blood DC populations are found CD11c myeloid DCs, which secrete IL 12, and CD123  [ **IL3RA** ]  IL 3  [ **IL3** ]  receptor DCs lymphoid, which secrete type I interferons in response to viral stimuli.  Ref: 11698285 Blood, 2001
- Cytokine R detectable on Ba but not MC, were interleukin 3  [ **IL3** ]  IL 3  [ **IL3** ]  R alpha CD123  [ **IL3RA** ] , IL 1RII CD121b, IL 2R alpha CD25 and CD40.  Ref: 8675206 Immunology, 1996
- Flow cytometric studies using primary AML tissue showed that the interleukin 3  [ **IL3** ]  receptor alpha chain IL 3Ralpha or CD123  [ **IL3RA** ]  was strongly expressed in CD34 CD38 cells 98 2% positive from 16 of 18 primary specimens.  Ref: 11021753 Leukemia, 2000
- The interleukin 3  [ **IL3** ]  IL 3  [ **IL3** ]  receptor CD123  [ **IL3RA** ]  was used to identify basophils in the leukocyte mixture.  Ref: 12587990 Homeopathy, 2003
- DESIGN AND METHODS We analyzed the expression of the interleukin 3  [ **IL3** ]  IL 3  [ **IL3** ]  receptor a chain CD123  [ **IL3RA** ]  in 22 normal samples and in a wide panel of hematologic malignancies using flow cytometry.  Ref: 11726317 Haematologica, 2001
- Although blast cells lacked basophil specific antigens such as CDw17, CD88, and FcepsilonRI, an expression profile of cytokine receptors including CD116 GM CSF receptor, CD117 c kit, and CD123  [ **IL3RA** ]  IL 3  [ **IL3** ]  receptor alpha helped to define the cellular lineage in our case.  Ref: 11999362 Int J Hematol, 2002
- Like normal basophils, basophils in MDS expressed interleukin 3  [ **IL3** ]  receptor alpha CD123  [ **IL3RA** ] , E NPP3 CD203c, CR1 CD35, CR3 CD11b, CR4 CD11c, membrane co factor protein CD46, decay accelerating factor CD55 and membrane attack complex inhibitory factor CD59, as well as receptors for C3a, C5a CD88, and IgE.  Ref: 11737228 Eur J Clin Invest, 2001
- Using a somatic cell hybrid mapping panel, we have assigned the mouse homolog of the human pseudoautosomal interleukin 3  [ **IL3** ]  receptor alpha subunit  **IL3RA**  gene to mouse Chromosome Chr 14.  Ref: 8903724 Mamm Genome, 1996
- According to their morphology, functions, and surface staining they could be separated into two distinct subpopulations mature HLA DRhi, mostly interleukin 3  [ **IL3** ]  R CD123  [ **IL3RA** ]  negative cells, associated with thymocytes, some apoptotic, and expressed myeloid and activation markers but no lymphoid markers.  Ref: 11129651 J Leukoc Biol, 2000
- Colony stimulating factor 2 receptor alpha CSF2RA and interleukin 3  [ **IL3** ]  receptor alpha  **IL3RA** , two genes from the chromosome Xp and Yp pseudoautosomal region PAR, have been suggested as candidate genes for short stature in Turner syndrome.  Ref: 8950669 J Med Genet, 1996
- These cells can be classified as basophils or mast cells using monoclonal antibodies mAbs against leukocyte antigens, including mast cell tryptase, c kit = mast cell growth factor  [ **IL3** ]  MGF receptor, interleukin 3  [ **IL3** ]  receptor alpha chain IL 3R alpha  [ **IL3RA** ]  = CD123  [ **IL3RA** ] , and CD11b C3biR.  Ref: 8819068 Leuk Lymphoma, 1996
- This was achieved based on simultaneous expression analysis of the monocyte markers CD14 and CD16 with antigens such as CD33, HLA DR, the integrin CD11c, and the interleukin 3  [ **IL3** ]  receptor alpha chain CD123  [ **IL3RA** ]  which in absence of lineage related antigens have been used for description of dendritic cell precursors.  Ref: 10879689 Immunobiology, 2000
- CD123  [ **IL3RA** ]  IL 3  [ **IL3** ]  receptor expression is similar in the three sources of hematopoietic cells at day 0 and after 48 h culture.  Ref: 10723580 Bone Marrow Transplant, 2000
- MATERIALS AND METHODS Based on data from 34 patients with hematologic malignancies, doses of CD34 cells and CD34 cell subsets, defined by the expression of HLA DR, CD38, CD117 c kit R, CD123  [ **IL3RA** ]  alpha subunit of IL 3  [ **IL3** ]  R, CD133 AC133, and CD90 Thy 1 antigens, were correlated with the number of short term i.e.  Ref: 11750108 Exp Hematol, 2001
